# Supplementary material for: Novel Organization of the Staphylococcal Cassette Chromosome mec Composite Island in Clinical Staphylococcus haemolyticus and Staphylococcus hominis Subspecies hominis Isolates from Dogs
Source: Microbiol Spectr. 2022 Jul 5;10(4):e00997-22. doi: 10.1128/spectrum.00997-22 (PMC9430635; doi:10.1128/spectrum.00997-22)
Supplement: Supplemental file 1 — Tables S1 to S4. Download spectrum.00997-22-s0001.pdf, PDF file, 0.3 MB [file spectrum.00997-22-s0001.pdf]

Supplemental materials

Table S1. Genetic features of SCC*mec* element in *S. haemolyticus* 1864

Gene	Position	Size (bp)	Function/ sequence	% similarity	% coverage	Reference strains	Accession no.
rlmH	28,790 - 29,269	480	Ribosomal RNA large subunit methyltransferase H	99	100	*Staphylococcus haemolyticus* strain 83131B	CP025396
DR1	29,252 - 29,269	18	GAAGCGTATCATAAATGA				
IR1	29,267 - 29,279	13	TGATGCGGTTTTT				
	29,505 - 30,122	618	Hypothetical protein	100	100	*Staphylococcus haemolyticus* strain 83131B	CP025396
cadC	30,141 - 30,482	342	Cadmium resistance transcriptional regulatory protein CadC	100	100	*Staphylococcus haemolyticus* strain 83131B	CP025396
arsC	30,502 - 30,903	402	Arsenate reductase	100	100	*Staphylococcus haemolyticus* strain 83131B	CP025396
arsB	30,921 - 32,210	1,290	Arsenical pump membrane protein	99.77	100	*Staphylococcus haemolyticus* strain VB5326	CP045137.2
8-bp identical sequence	32,593 - 32,600	8	CTTTTTGC				
IS*431*	32,657 - 33,331	675	IS6 family transposase IS431	100	100	*Staphylococcus epidermidis* strain NBRC113846	CP084008

Table S1 (Continued)

Gene	Position	Size (bp)	Function/ sequence	% similarity	% coverage	Reference strains	Accession no.
mecA	33,507 - 35,513	2,007	PBP2a family beta-lactam-resistant peptidoglycan transpeptidase MecA	100	100	*Staphylococcus haemolyticus* strain VB5326	CP045137.2
paaZ	35,559 - 35,987	429	Bifunctional protein PaaZ	100	100	*Staphylococcus aureus* strain TPS5614	AP025176.1
ugpQ	36,084 - 36,827	744	Glycerophosphodiester phosphodiesterase	100	100	*Staphylococcus aureus* strain TPS5614	AP025176.2
	37,744 - 37,911	168	Hypothetical protein	100	100	*Staphylococcus aureus* strain TPS5614	AP025176.3
8-bp identical sequence	38,903-38,910	8	CTTTTTGC				
IS*431*	38,169 - 38,843	675	IS6 family transposase IS431	100	100	*Staphylococcus epidermidis* strain NBRC113846	CP084008.1
copA	39,106 - 41,166	2,061	CopA, Copper-exporting P-type ATPase A	100	100	*Staphylococcus haemolyticus* strain VB5326	CP045137.2
ydhk	41,184 - 41,729	546	Putative protein YdhK	100	100	*Staphylococcus haemolyticus* strain VB5326	CP045137.2
	42,017 - 42,694	678	Hypothetical protein	100	100	*Staphylococcus haemolyticus* strain VB5326	CP045137.3

Table S1 (Continued)

Gene	Position	Size (bp)	Function/ sequence	% similarity	% coverage	Reference strains	Accession no.
IR2	41,908 - 41,920	13	AAAAACCGCATCA				
DR2	41,927- 41,952	18	GAA GCA TAT CAT AAG TGA				
ISSep*1*	44,593 - 42,974	1,620	IS1182 family transposase ISSep1	100	100	*Staphylococcus haemolyticus* strain 83131A	CP024809.1
	45,253 - 45,489	237	Hypothetical protein	99.58	100	*Staphylococcus haemolyticus* strain VB5326	CP045137.2
yiaC	45,657 - 46,091	435	Peptidyl-lysine N-acetyltransferase YiaC	99.58	100	*Staphylococcus haemolyticus* strain VB5326	CP045137.2
merR	46,587 - 46,934	348	MerR family transcriptional regulator	100	100	*Staphylococcus haemolyticus* strain VB5326	CP045137.2
hchA	47,012 - 47,695	684	Protein/nucleic acid deglycase HchA	100	100	*Staphylococcus haemolyticus* strain VB5326	CP045137.2
yhfK	47,717 - 48,382	666	Putative sugar epimerase YhfK	100	100	*Staphylococcus haemolyticus* strain VB5326	CP045137.2
yfmJ	48,386 - 49,390	1,005	Putative NADP-dependent oxidoreductase YfmJ	100	100	*Staphylococcus haemolyticus* strain VB5326	CP045137.2
	49,399 - 49,764	366	Hypothetical protein	100	100	*Staphylococcus haemolyticus* strain VB5326	CP045137.2

Table S1 (Continued)

Gene	Position	Size (bp)	Function/ sequence	% similarity	% coverage	Reference strains	Accession no.
	50,286 - 50,903	618	Hypothetical protein	100	100	*Staphylococcus haemolyticus* strain VB5326	CP045137.2
cadC	50,922 - 51,263	342	Cadmium resistance transcriptional regulatory protein CadC	100	100	*Staphylococcus haemolyticus* strain VB19458	CP045187.2
arsC	51,283 - 51,684	402	Arsenate reductase	100	100	*Staphylococcus haemolyticus* strain VB19458	CP045187.2
arsB	51,702 - 52,994	1,293	Arsenical pump membrane protein	100	100	*Staphylococcus haemolyticus* strain VB19458	CP045187.2
sdpR	52,994 - 53,308	315	Transcriptional repressor SdpR	100	100	*Staphylococcus haemolyticus* strain VB5326	CP045137.2
IR3	53,403- 53,415	13	AAAAAACGCATCA				
	53,646 - 53,981	336	Hypothetical protein	100	100	*Staphylococcus haemolyticus* strain VB5326	CP045137.2
	53,999 - 54,193	195	Hypothetical protein	100	100	*Staphylococcus haemolyticus* strain SCAID URN1-2019	

Table S1 (Continued)

Gene	Position	Size (bp)	Function/ sequence	% similarity	% coverage	Reference strains	Accession no.
	54,190 - 54,357	168	Hypothetical protein	100	100	*Staphylococcus haemolyticus* strain SCAID URN1-2019	CP052055.1
mscS	55,435 - 54,548	888	Small-conductance mechanosensitive channel	100	100	*Staphylococcus haemolyticus* strain SCAID URN1-2019	CP052055.1
	55,669 - 55,857	189	Hypothetical protein	100	100	*Staphylococcus haemolyticus* strain VB5326	CP045137.2
	55,944 - 56,468	525	Hypothetical protein	100	100	*Staphylococcus haemolyticus* strain VB19458	CP045187.2
	56,542 - 57,420	879	Hypothetical protein	100	100	*Staphylococcus haemolyticus* strain VB19458	CP045187.2
znuA	57,687 - 58,670	984	High-affinity zinc uptake system binding-protein ZnuA	100	100	*Staphylococcus haemolyticus* strain SCAID URN1-2019	CP052055.1
	58,849 - 58,971	123	Hypothetical protein	100	100	*Staphylococcus haemolyticus* strain VB5326	CP045137.2

Table S1 (Continued)

Gene	Position	Size (bp)	Function/ sequence	% similarity	% coverage	Reference strains	Accession no.
	59,221 - 60,648	1,428	Hypothetical protein	100	100	*Staphylococcus haemolyticus* strain VB5326	CP045137.2
azoR1	60,740 - 61,036	297	FMN-dependent NADH-azoreductase 1	100	100	*Staphylococcus haemolyticus* strain SCAID URN1-2019	CP052055.1
	61,477 - 61,602	126	Hypothetical protein	100	100	*Staphylococcus haemolyticus* strain SCAID URN1-2019	CP052055.1
dapE	61,983 - 63,206	1,224	Putative succinyl-diaminopimelate desuccinylase	100	100	*Staphylococcus haemolyticus* strain VB19458	CP045187.2
	63,509 - 64,561	1,053	Hypothetical protein	100	100	*Staphylococcus haemolyticus* strain VB5326	CP045137.2
thrZ	64,984 - 66,900	1,917	Threonine-tRNA ligase 2	100	100	*Staphylococcus haemolyticus* strain VB19458	CP045187.2
btuD	67,091 - 67,912	822	Vitamin B12 import ATP-binding protein BtuD	100	100	*Staphylococcus haemolyticus* strain VB5326	CP045137.2
	67,899 - 68,054	156	Hypothetical protein	97	64	*Staphylococcus aureus* strain R46	CP039164.1

Table S1 (Continued)

Gene	Position	Size (bp)	Function/ sequence	% similarity	% coverage	Reference strains	Accession no.
IS*256*	68,025 - 69,197	1,173	IS256 family transposase IS256	100	100	*Staphylococcus aureus* TPS5614	AP025176.1
	69,348 - 69,935	588	Hypothetical protein	100	100	*Staphylococcus haemolyticus* strain SCAID URN1-2019	CP052055.1
	69,987 - 70,181	195	Hypothetical protein	100	100	*Staphylococcus haemolyticus* strain SCAID PHRX1-2019	CP052056.1
ISSha*1*	70,168 - 71,484	1,317	ISL3 family transposase ISSha1	100	100	*Staphylococcus haemolyticus* strain VB5326	CP045137.2
	71,670 - 71,942	237	Hypothetical protein	100	100	*Staphylococcus haemolyticus* strain VB5326	CP045137.2
	71,999 - 72,316	318	Hypothetical protein	100	100	*Staphylococcus haemolyticus* strain VB5326	CP045137.2
icaC	72,819 - 73,886	1,068	Putative poly-beta-1,6-N-acetyl-D-glucosamine export protein	100	100	*Staphylococcus haemolyticus* strain Sh29/312/L2	CP011116.1

Table S1 (Continued)

Gene	Position	Size (bp)	Function/ sequence	% similarity	% coverage	Reference strains	Accession no.
mntC	76,154 - 77,083	930	ABC superfamily ATP binding cassette transporter, membrane protein	100	100	*Staphylococcus haemolyticus* strain Sh29/312/L2	CP011116.1
mntB	77,080 - 77,916 -	837	ABC superfamily ATP binding cassette transporter, membrane protein	100	100	*Staphylococcus haemolyticus* strain VB19458	CP045187.2
mntA	77,909 - 78,652	744	ABC superfamily ATP binding cassette transporter, membrane protein	99.87	100	*Staphylococcus haemolyticus* strain Sh29/312/L2	CP011116.1
yciC	78,911 - 80,110	1,200	Putative metal chaperone YciC	99.67	100	*Staphylococcus haemolyticus* strain SCAID URN1-2019	CP052055.1
brnQ	80,336 - 81,697	1,362	Branched-chain amino acid transport system 2 carrier protein	100	100	*Staphylococcus haemolyticus* strain VB19458	CP045187.2
cmoB	82,053 - 82,745	693	tRNA U34 carboxymethyltransferase	100	100	*Staphylococcus haemolyticus* strain Sh29/312/L2	CP011116.1

Table S2. Genetic features of SCC*mec* element in *S. haemolyticus* 48

Gene	Position	Size (bp)	Function/ sequence	% similarity	% coverage	Reference strains	Accession no.
rlmH	28,796 - 29,275	480	Ribosomal RNA large subunit methyltransferase H	99.58	100	*Staphylococcus haemolyticus* strain SCAID PHRX1-2019	CP052056.1
DR1	29,258 - 29,275	18	GAAGCATATCACAAATAA				
IS1	29,261-29,268	8	GCATATCA				
tri1	29,404 - 30,390	987	ADP-ribosylarginine hydrolase Tri1	100	100	*Staphylococcus haemolyticus* strain SCAID PHRX1-2019	CP052055.1
	30,409 - 31,743	1,335	Hypothetical protein	100	100	*Staphylococcus haemolyticus* strain VB19458	CP045187.2
ydjH	31,740 - 32,678	939	Putative sugar kinase YdjH	100	100	*Staphylococcus haemolyticus* strain SH32	KF006347.1
IS*431*	32,716 - 33,390	675	IS6 family transposase IS431	100	100	*Staphylococcus haemolyticus* strain FDAARGOS_517	CP033814
	34,194 - 33,847	348	Hypothetical protein	100	100	*Staphylococcus haemolyticus* strain FDAARGOS_517	CP033814

Table S2 (Continued)

Gene	Position	Size (bp)	Function/ sequence	% similarity	% coverage	Reference strains	Accession no.
hchA	34,272 - 34,955	684	Protein/nucleic acid deglycase HchA	100	100	*Staphylococcus haemolyticus* strain VB5326	CP045137.2
yhfK	34,977 - 35,642	666	Putative sugar epimerase YhfK	100	100	*Staphylococcus haemolyticus* strain SCAID URN1-2019	CP052055.1
yfmJ	35,646 - 36,650	1,005	Putative NADP-dependent oxidoreductase YfmJ	100	100	*Staphylococcus haemolyticus* strain SCAID URN1-2019	CP052055.1
	36,659 - 37,024	366	Hypothetical protein	100	100	*Staphylococcus haemolyticus* strain SCAID URN1-2019	CP052055.1
cadC	37,560 - 38,177	618	Hypothetical protein	100	100	*Staphylococcus haemolyticus* strain PK-01	CP035541.1
cadX	38,557 - 38,958	342	Cadmium resistance transcriptional regulatory protein CadC	100	100	*Staphylococcus haemolyticus* strain WCH1	JQ764731.1
arsC	38,557 - 38,958	402	Arsenate reductase	100	100	*Staphylococcus haemolyticus* strain SCAID URN1-2019	CP052055.1

Table S2 (Continued)

Gene	Position	Size (bp)	Function/ sequence	% similarity	% coverage	Reference strains	Accession no.
arsB	38,976 - 40,265	1,290	Arsenical pump membrane protein	100	100	*Staphylococcus haemolyticus* strain SH32	KF006347.1
arsR	40,265 - 40,582	318	Arsenical resistance operon repressor	100	100	*Staphylococcus haemolyticus* strain SCAID URN1-2019	CP052055.1
IS*431*	40,712 - 41,386	675	IS6 family transposase IS431	100	100	*Staphylococcus epidermidis* strain NBRC 113846	CP084008.1
mecA	41,562 - 43,568	2,007	PBP2a family beta-lactam-resistant peptidoglycan transpeptidase MecA	100	100	*Staphylococcus haemolyticus* strain VB5326	CP045137.2
paaZ	43,614 - 44,042	429	Bifunctional protein PaaZ	100	100	*Staphylococcus aureus* strain TPS5614	AP025176.1
ugpQ	44,139 - 44,882	744	Glycerophosphodiester phosphodiesterase	100	100	*Staphylococcus aureus* strain TPS5614	AP025176.1
	45,399 - 45,566	168	Hypothetical protein	100	100	*Staphylococcus aureus* strain TPS5614	AP025176.1
IS*431*	45,824 - 46,498	675	IS6 family transposase IS431	100	100	*Staphylococcus epidermidis* strain NBRC 113846	CP084008.1

Table S2 (Continued)

Gene	Position	Size (bp)	Function/ sequence	% similarity	% coverage	Reference strains	Accession no.
copA	46,761 - 48,821	2,061	Copper-exporting P-type ATPase B	100	100	*Staphylococcus haemolyticus* strain SH32	KF006347.1
mco	48,836 - 50,269	1,434	Multicopper oxidase	100	100	*Staphylococcus haemolyticus* strain K8	LT963441.1
ydhk	50,289 - 50,771	483	Putative protein YdhK	100	100	*Staphylococcus haemolyticus* strain WCH1	JQ764731.1
arsC	50,977 - 51,372	396	Arsenate reductase	100	100	*Staphylococcus haemolyticus* strain SH32	KF006347.1
arsB	51,391 - 52,683	1,293	Arsenical pump membrane protein	100	100	*Staphylococcus haemolyticus* strain SH32	KF006347.1
	52,683 - 52,997	315	Hypothetical protein	100	100	*Staphylococcus haemolyticus* strain K8	LT963441.1
	52,994 - 53,137	144	Hypothetical protein	100	100	*Staphylococcus haemolyticus* strain K8	LT963441.1
arsA	53,137 - 54,867	1,731	Arsenical pump-driving ATPase	100	100	*Staphylococcus haemolyticus* strain SH32	KF006347.1

Table S2 (Continued)

Gene	Position	Size (bp)	Function/ sequence	% similarity	% coverage	Reference strains	Accession no.
arsD	54,848 - 55,195	348	Arsenical resistance operon trans-acting repressor ArsD	100	100	*Staphylococcus haemolyticus* strain SH32	KF006347.1
	55,478 - 55,672	195	Hypothetical protein	100	100	*Staphylococcus haemolyticus* strain SH32	KF006347.1
arsR	55,718 - 56,038	321	Arsenical resistance operon repressor	100	100	*Staphylococcus haemolyticus* strain SH32	KF006347.2
	56,126 - 57,010	885	Putative two-component membrane permease complex subunit SMU_747c	100	100	*Staphylococcus haemolyticus* strain NW19A	KM369884.1
	57,024 - 57,152	129	Hypothetical protein	100	100	*Staphylococcus hominis* strain FDAARGOS_762	CP054006.1
IR2	57,302 - 57,328	13	AAAAACCGCATCA				
DR2	57,329 - 57,346	18	GAAGCGTATCATAAATAA				
	59,827 - 57,878	1,950	Type III restriction endonuclease	100	100	*Staphylococcus haemolyticus* strain 83131B	CP025396.1
	59,829 - 62,798	2,970	Type III restriction-modification system endonuclease	99.97	100	*Staphylococcus haemolyticus* strain 83131B	CP025396.1

Table S2 (Continued)

Gene	Position	Size (bp)	Function/ sequence	% similarity	% coverage	Reference strains	Accession no.
	62,808 - 64,097	1,290	Helicase	100	100	*Staphylococcus haemolyticus* strain 83131B	CP025396.1
	64,081 - 64,512	432	Hypothetical protein	100	98	*Staphylococcus haemolyticus* strain 83131B	CP025396.1
IS*256*	64,609 - 65,781	1,173	IS256 family transposase IS256	100	100	*Staphylococcus aureus* strain TPS5614	AP025176.1
	65,826 - 66,230	405	Hypothetical protein	100	100	*Enterococcus faecium* strain 4995/20 plasmid p4995_1	CP084179
aph(2")-Ia	66,231 - 67,670	1,440	aminoglycoside O-phosphotransferase APH(2")-Ia	100	100	*Staphylococcus aureus* strain UMCG578	CP077738.1
IS*256*	67,800 - 68,972	1,173	IS256 family transposase IS256	100	100	*Staphylococcus aureus* strain TPS5614	AP025176.1
	69,087 - 70,529	1,443	Hypothetical protein	100	100	*Staphylococcus haemolyticus* strain 83131B	CP025396.1
	70,850 - 70,981	132	Hypothetical protein	100	100	*Staphylococcus haemolyticus* strain SH32	KF006347.1
	70,996 - 71,499	504	Hypothetical protein	99.4	100	*Staphylococcus haemolyticus* strain 83131B	CP025396.1

Table S2 (Continued)

Gene	Position	Size (bp)	Function/ sequence	% similarity	% coverage	Reference strains	Accession no.
	71,515 - 71,826	312	Hypothetical protein	100	100	*Staphylococcus haemolyticus* strain 83131B	CP025396.1
	71,828 - 71,917	90	Hypothetical protein	100	100	*Staphylococcus haemolyticus* strain 83131B	CP025396.1
	71,919 - 72,260	342	Hypothetical protein	100	100	*Staphylococcus haemolyticus* strain 83131B	CP025396.1
nupC	72,820 - 74,001	1,182	Nucleoside permease NupC	99.92	100	*Staphylococcus aureus* subsp. *aureus* strain M06/0171	HE980450.1
psuG	74,012 - 74,923	912	Pseudouridine-5'-phosphate glycosidase	99.78	100	*Staphylococcus haemolyticus* strain 83131B	CP025396.1
	74,907 - 76,016	1,109	Ribokinase	99.91	100	*Staphylococcus haemolyticus* strain 83131B	CP025396.1
ccrB4	76,173 - 77,798	1,626	Cassette chromosome recombinase B4	100	100	*Staphylococcus haemolyticus* strain 06	LS483319.1
ccrA4	77,795 - 78,271	477	Cassette chromosome recombinase A4	99.79	100	*Staphylococcus aureus* subsp. *aureus* strain M06/0171	HE980450.1

Table S2 (Continued)

Gene	Position	Size (bp)	Function/ sequence	% similarity	% coverage	Reference strains	Accession no.
ccrA4	78,276 - 79,157	882	Cassette chromosome recombinase A4	99.32	100	*Staphylococcus aureus* subsp. *aureus* strain M06/0171	HE980450.1
moxC	79,412 - 80,704	1,293	Putative monooxygenase MoxC	100	100	*Staphylococcus aureus* subsp. *aureus* strain M06/0171	HE980450.1
DR3	80,734 - 80,751	18	GAAGCGTATCACAAATAA				
IR3	80,737-80,740	8	GCGTATCA				
lagD	81,300 - 83,462	2,163	Lactococcin-G-processing and transport ATP-binding protein LagD	77.93	94	*Staphylococcus haemolyticus* ATCC 29970 plasmid unnamed	CP035292.1
	83,543 - 83,659	117	Hypothetical protein	87.93	99	*Staphylococcus epidermidis* strain NCCP 16828	CP043847.1
	83,689-83,871	183	Hypothetical protein	92.16	27	*Staphylococcus epidermidis* strain NCCP 16828	CP043847.1

Table S2 (Continued)

Gene	Position	Size (bp)	Function/ sequence	% similarity	% coverage	Reference strains	Accession no.
IR4	85,275 - 85,282	8	GATAGGGA				
DR4	85,283 - 85,300	18	GAAGCATATCACAAATAA				
	85,366 - 86,049	684	Hypothetical protein	100	100	*Staphylococcus aureus* strain PTDrAP2	CP029172.1
	86,439 - 87,098	660	Hypothetical protein	99.55	100	*Staphylococcus aureus* strain PTDrAP2	CP029172.1
gph	87,234 - 88,598	1,365	Phosphoglycolate phosphatase	99.71	100	*Staphylococcus aureus* strain PTDrAP2	CP029172.1
tarS	89,406 - 89,663	258	Poly(ribitol-phosphate) beta-N-acetylglucosaminyltransferase TarS	93	100	*Staphylococcus aureus* strain PTDrAP2	CP029172.1
IS*431*	89,704 - 90,378	675	IS6 family transposase IS431	100	100	*Staphylococcus haemolyticus* strain S167	CP013911.1
tet(K)	90,575 - 91,954	1,380	Tetracycline efflux MFS transporter Tet(K)	100	100	*Staphylococcus saprophyticus* strain UTI-056 plasmid pUTI-056-3	CP054447.1

Table S2 (Continued)

Gene	Position	Size (bp)	Function/ sequence	% similarity	% coverage	Reference strains	Accession no.
	92,140 - 93,381	1,274	Hypothetical protein	100	100	*Staphylococcus saprophyticus* strain UTI-056 plasmid pUTI-056-3	CP054447.1
	93,567 - 93,668	102	Hypothetical protein	100	100	*Staphylococcus saprophyticus* strain UTI-056 plasmid pUTI-056-3	CP054447.1
repN	94,023 - 94,862	840	Replication initiation protein	100	100	*Staphylococcus hominis* strain FDAARGOS_762	CP054006.1
IS*431*	94,950 - 95,624	675	IS6 family transposase IS431	100	100	*Staphylococcus haemolyticus* strain SH32	KF006347.1

Table S3. Genetic features of SCC*mec* element in *S. hominis* subsp. *hominis* 384

Gene	Position	Size (bp)	Function/ sequence	% similarity	% coverage	Reference strains	Accession no.
rlmH	28,031 - 28,510	480	Ribosomal RNA large subunit methyltransferase H	97.69	99	*Staphylococcus hominis* strain J6	LT963442.1
DR1	28,493 - 28,510	18	GAAGCTTATCATAAGTAA				
IR1	28,511 - 28,527	17	TGAGGTTCATGATTTTT				
	28,730 - 29,986	1,257	Hypothetical protein	100	100	*Staphylococcus epidermidis* strain NBRC 113846	CP084008.1
ISSep*1*	30,081 - 31,769	1,689	IS1182 family transposase ISSep1	99.76	98	*Staphylococcus hominis* strain FDAARGOS_745	CP050982.1
	32,311 - 32,553	243	Hypothetical protein	100	100	*Staphylococcus aureus* strain ER04164.3	CP030542.1
IS*257*	32,581 - 33,255	675	IS6 family transposase IS257R2	100	100	*Staphylococcus hominis* strain FDAARGOS_661 plasmid unnamed3	CP054552.1
thyA	33,355 - 34,311	957	Thymidylate synthase	100	100	*Staphylococcus saprophyticus* UTI-058y plasmid pUTI-058y-1	CP054441.1
dfrC	34,353 - 34,838	486	Trimethoprim-resistant dihydrofolate reductase	100	100	*Staphylococcus saprophyticus* UTI-058y plasmid pUTI-058y-1	CP054441.1

Table S3 (Continued)

Gene	Position	Size (bp)	Function/ sequence	% similarity	% coverage	Reference strains	Accession no.
	34,848 - 35,270	423	DegV domain-containing protein	100	100	*Staphylococcus saprophyticus* UTI-058y plasmid pUTI-058y-1	CP054441.1
IS*257*	35,302 - 35,976	675	IS6 family transposase IS257R1	100	100	*Staphylococcus aureus* strain ER04164.3	CP030542.1
	36,234 - 36,401	168	Hypothetical protein	100	100	*Staphylococcus aureus* strain TPS5614	AP025176.1
ugpQ	37,278 - 38,021	744	Glycerophosphoryl diester phosphodiesterase	100	100	*Staphylococcus aureus* strain TPS5614	AP025176.1
paaZ/ maoC	38,118 - 38,546	429	Bifunctional protein PaaZ/ MaoC family dehydratase	100	100	*Staphylococcus aureus* strain TPS5614	AP025176.1
mecA	38,592 - 40,598	2,007	PBP2a family beta-lactam-resistant peptidoglycan transpeptidase MecA	100	100	*Staphylococcus aureus* strain TPS5614	AP025176.1
mecR1	40,698 - 42,455	1,758	Methicillin resistance mecR1 protein	100	100	*Staphylococcus hominis* strain FDAARGOS_748	CP054883.1
mecI	42,455 - 42,826	372	Methicillin resistance regulatory protein MecI	100	100	*Staphylococcus epidermidis* strain B1200343	CP073821.1
IS*431*	43,075 - 43,749	675	IS6 family transposase IS431	100	100	*Staphylococcus aureus* strain TPS3156	AP023034.1

Table S3 (Continued)

Gene	Position	Size (bp)	Function/ sequence	% similarity	% coverage	Reference strains	Accession no.
	44,104 - 44,445	342	Hypothetical protein	100	100	*Staphylococcus hominis* strain FDAARGOS_661	CP054550.1
gloB	44,714 - 46,042	1,329	Hydroxyacylglutathione hydrolase	99.02	100	*Staphylococcus hominis* strain J6	LT963442.1
glpE	46,061 - 47,128	1,068	Thiosulfate sulfurtransferase GlpE	100	100	*Staphylococcus haemolyticus* strain 12b	CP071505.1
	47,267 - 47,527	261	Hypothetical protein	100	100	*Staphylococcus haemolyticus* strain 12b	CP071505.1
	47,524 - 48,282	759	Hypothetical protein	99.87	100	*Staphylococcus hominis* strain FDAARGOS_745	CP050982.1
copB	48,494 - 48,661	168	copper P-type ATPase AtkB	99.4	100	*Staphylococcus haemolyticus* strain SCAID URN1-2019	CP052055.1
ydhK	49,224 - 48,679	546	Putative protein YdhK	99.63	100	*Staphylococcus aureus* strain ER03868.3	CP030403.1
IR2	49,403 - 49,415	13	AAAAACCGCATCA				
DR2	49,430 - 49,447	18	GAAGCATATCATAAATAA				
apbE	51,031 - 50,114	918	FAD:protein FMN transferase	100	100	*Staphylococcus hominis* strain C34847	CP014567.1

Table S3 (Continued)

Gene	Position	Size (bp)	Function/ sequence	% similarity	% coverage	Reference strains	Accession no.
namA	51,114 - 54,131	3,018	NADPH dehydrogenase	99.83	100	*Staphylococcus hominis* strain 19A	CP031277.1
norB	54,153 - 55,532	1,380	Quinolone resistance protein NorB	99.93	100	*Staphylococcus hominis* strain 19A	CP031277.1
budC	55,908 - 55,723	186	L-2,3-butanediol dehydrogenase	99.46	100	*Staphylococcus hominis* strain C34847	CP031277.1
speG	56,283 - 56,780	498	Spermidine N(1)-acetyltransferase	99	100	*Staphylococcus hominis* strain 19A	CP014567.1
	58,190 - 58,972	783	Hypothetical protein	97.96	100	*Staphylococcus hominis* strain C34847	CP031277.1
	59,328 - 59,711	384	Hypothetical protein	99.74	100	*Staphylococcus hominis* strain FDAARGOS_745	CP014567.1
	59,723 - 60,355	633	Hypothetical protein	100	100	*Staphylococcus hominis* strain FDAARGOS_745	CP050982.1
arsB	60,908 - 62,200	1,293	Arsenical pump membrane protein	100	100	*Staphylococcus hominis* strain FDAARGOS_745	CP050982.1
	62,200 - 62,514	315	Hypothetical protein	100	100	*Staphylococcus hominis* strain FDAARGOS_745	CP050982.1
cdr	62,511 - 64,175	1,665	Coenzyme A disulfide reductase	100	100	*Staphylococcus hominis* strain FDAARGOS_745	CP050982.1

Table S3 (Continued)

Gene	Position	Size (bp)	Function/ sequence	% similarity	% coverage	Reference strains	Accession no.
arsA	64,175 - 65,902	1,728	Arsenical pump-driving ATPase	100	100	*Staphylococcus hominis* strain FDAARGOS_745	CP050982.1
arsD	65,883 - 66,230	348	Arsenical resistance operon trans-acting repressor ArsD	99.71	100	*Staphylococcus hominis* strain 19A	CP031277.1
	66,512 - 66,706	195	Hypothetical protein	99.49	100	*Staphylococcus hominis* strain FDAARGOS_748	CP054883.1
arsR	66,752 - 67,072	321	Arsenical resistance operon repressor	100	100	*Staphylococcus hominis* strain FDAARGOS_661	CP054550.1
	67,160 - 68,044	885	Putative two-component membrane permease complex subunit SMU_747c	99.77	100	*Staphylococcus hominis* strain C34847	CP014567.1
	68,058 - 68,186	129	Hypothetical protein	99.22	100	*Staphylococcus warneri* strain 16A plasmid unnamed3	CP031267.1
IR3	68,337 - 68,349	13	AAAAACCGCATCA				
copA	68,897 - 70,960	2,064	Copper-exporting P-type ATPase B	99.76	100	*Staphylococcus hominis* strain 19A	CP031277.1
mco	70,975 - 72,408	1,434	Multicopper oxidase mco	100	100	*Staphylococcus hominis* strain 19A	CP031277.1

Table S3 (Continued)

Gene	Position	Size (bp)	Function/ sequence	% similarity	% coverage	Reference strains	Accession no.
ydhK	72,486 - 72,815	330	Putative protein YdhK	100	100	*Staphylococcus hominis* strain 19A	CP031277.1
IR4	72,994 - 73,007	13	AAAAACCGCATCA				
DR3	73,021- 73,038	18	GACGCTTATCATAAGTGA				

Table S4. Genetic features of SCC*mec* element in *S. hominis* subsp. *hominis* 371

Gene	Position	Size (bp)	Function/ sequence	% similarity	% coverage	Reference strains	Accession no.
rlmH	28,031 - 28,510	480	Ribosomal RNA large subunit methyltransferase H	97.08	100	*Staphylococcus hominis* strain FDAARGOS_747	CP046301.1
DR1	28,493- 28,510	18	GAA GCG TAC CAC AAA TAA				
	29,031 - 30,776	1,746	Hypothetical protein	99.96	100	*Staphylococcus aureus* strain 628	CP022905.1
	31,383 - 31,556	174	Hypothetical protein	84.12	97	*Staphylococcus delphini* strain NCTC12225	LR134263.1
	32,531 - 32,265	267	Hypothetical protein	98.84	32	*Staphylococcus haemolyticus* strain VB19458	CP045187.2
	32,491 - 32,712	222	Hypothetical protein	94.59	100	*Staphylococcus hominis* subsp. *hominis* strain NTUH-3390	KY643657.1
	32,726 - 33,121	387	DUF1643 domain-containing protein	93.28	100	*Staphylococcus saprophyticus* strain 1A	CP031196.1
	33,246 - 33,557	312	Hypothetical protein	97.76	100	*Staphylococcus aureus* strain 14505	CP053640.1
	33,644 - 33,994	351	Hypothetical protein	100	100	*Staphylococcus auricularis* strain NCTC12101	LS483491.1

Table S4 (Continued)

Gene	Position	Size (bp)	Function/ sequence	% similarity	% coverage	Reference strains	Accession no.
	34,535 - 35,152	618	Hypothetical protein	95.16	100	*Staphylococcus lugdunensis* strain JICS135	AP021848.1
	35,713 - 35,204	510	Hypothetical protein	98.71	100	*Staphylococcus hominis* strain 19A	CP031277.1
zipA	35,710 - 36,282	573	Cell division protein ZipA	98.82	100	*Staphylococcus hominis* strain FDAARGOS_136	CP014107.1
	36,297 - 36,824	528	Hypothetical protein	84.43	100	*Staphylococcus hominis* strain FDAARGOS_136	CP014107.1
ccrB4	36,948 - 38,576	1,629	Cassette chromosome recombinase B4	99.63	100	*Staphylococcus aureus* strain AR466	CP029080.1
ccrA4	38,573 - 39,934	1,362	Cassette chromosome recombinase A4	100	100	*Staphylococcus aureus* strain AR466	CP029080.1
	40,145 - 41,911	1,767	Hypothetical protein	100	100	*Staphylococcus aureus* strain AR466	CP029080.1
	41,911 - 42,204	294	Hypothetical protein	100	100	*Staphylococcus aureus* strain AR466	CP029080.1
ISSep*1*	42,323 - 44,002	1,680	IS1182 family transposase ISSep1	99.82	100	*Staphylococcus hominis* strain 19A	CP031277.1

Table S4 (Continued)

Gene	Position	Size (bp)	Function/ sequence	% similarity	% coverage	Reference strains	Accession no.
	44,286 - 45,800	1,515	Hypothetical protein	100	100	*Staphylococcus epidermidis* strain Z0118SE0132	CP061029
	47,342 - 46,431	912	Putative thiamine biosynthesis protein	100	100	*Staphylococcus aureus* strain AR466	CP029080.1
IS*431*	47,592 - 48,266	675	IS6 family transposase IS431	100	100	*Staphylococcus aureus* strain P5	CP092475
	44,286 - 45,800	168	Hypothetical protein	100	100	*Staphylococcus aureus* strain TPS5614	AP025176.1
ugpQ	49,608 - 50,351	744	Glycerophosphodiester phosphodiesterase, cytoplasmic	100	100	*Staphylococcus aureus* strain TPS5614	AP025176.1
paaZ	50,448 - 50,876	429	Bifunctional protein PaaZ	100	100	*Staphylococcus aureus* strain TPS5614	AP025176.1
mecA	50,922 - 52,928	2,007	PBP2a family beta-lactam-resistant peptidoglycan transpeptidase MecA	100	100	*Staphylococcus aureus* strain TPS5614	AP025176.1
mecR1_1	53,028 - 53,840	813	Methicillin resistance mecR1 protein	99	100	*Staphylococcus aureus* strain TPS5614	AP025176.1
mecR1_2	53,872 - 54,786	915	Methicillin resistance mecR1 protein	100	100	*Staphylococcus hominis* strain FDAARGOS_748	CP054883.1

Table S4 (Continued)

Gene	Position	Size (bp)	Function/ sequence	% similarity	% coverage	Reference strains	Accession no.
mecI	54,786 - 55,157	372	Methicillin resistance regulatory protein MecI	100	100	*Staphylococcus epidermidis* strain B1200343	CP073821.1
nagC	55,630 - 56,778	1,149	N-acetylglucosamine repressor	100	100	*Staphylococcus aureus* strain JRA307	AP019751.1
glpE	56,892 - 57,197	306	Thiosulfate sulfurtransferase GlpE	100	100	*Staphylococcus epidermidis* strain B1200343	CP073821.1
	57,925 - 58,308	384	Hypothetical protein	99.7	100	*Staphylococcus hominis* strain C34847	CP014567.1
	58,320 - 58,952	633	Hypothetical protein	99.37	100	*Staphylococcus hominis* strain FDAARGOS_745	CP050982.1
arsC	59,091 - 59,486	396	Arsenate reductase	97.47	100	*Staphylococcus hominis* strain FDAARGOS_745	CP050982.1
arsB	59,505 - 60,794	1,290	Arsenical pump membrane protein	97.29	100	*Staphylococcus hominis* strain FDAARGOS_661	CP054550.1
	60,797 - 61,111	315	Hypothetical protein	100	100	*Staphylococcus hominis* strain FDAARGOS_661	CP054550.1
arsA	61,251-62,981	1731	Arsenical pump-driving ATPase	99.42	100	*Staphylococcus hominis* strain FDAARGOS_746	CP046306.1

Table S4 (Continued)

Gene	Position	Size (bp)	Function/ sequence	% similarity	% coverage	Reference strains	Accession no.
arsD	62,962 - 63,309	348	Arsenical resistance operon trans-acting repressor ArsD	99.71	100	*Staphylococcus hominis* strain FDAARGOS_762	CP054006.1
	63,591 - 63,785	195	Hypothetical protein	98.97	100	*Staphylococcus hominis* strain FDAARGOS_661	CP054550.1
arsR	63,831 - 64,151	321	Arsenical resistance operon repressor	98.44	100	*Staphylococcus hominis* strain FDAARGOS_661	CP054550.2
	64,239 - 65,123	885	Putative two-component membrane permease complex subunit SMU_747c	96.95	100	*Staphylococcus hominis* strain 19A	CP031277.1
	65,137 - 65,265	129	Hypothetical protein	100	100	*Staphylococcus hominis* strain FDAARGOS_762	CP054006.1
IR1	65,416 - 65428	13	AAAAACCGTATCA				
copA	65,976 - 68,039	2,064	Copper-exporting P-type ATPase B	99.08	100	*Staphylococcus hominis* strain FDAARGOS_746	CP046306.1
ydhk	68,057 - 68,602	546	Putative protein YdhK	99.82	100	*Staphylococcus hominis* strain FDAARGOS_748	CP054883.1
	68,864 - 69,331	468	Hypothetical protein	99.79	100	Staphylococcus hominis FDAARGOS_747	CP046301.1

Table S4 (Continued)

Gene	Position	Size (bp)	Function/ sequence	% similarity	% coverage	Reference strains	Accession no.
	69,880 - 71,031	1,152	Putative zinc-binding alcohol dehydrogenase	98.96	100	*Staphylococcus hominis* strain 19A	CP031277.1
	71,734 - 72,273	540	Hypothetical protein	99.81	100	*Staphylococcus hominis* strain 19A	CP031277.1
adhA	72,588 - 73,628	1,041	Putative formaldehyde dehydrogenase AdhA	98.94	100	*Staphylococcus hominis* strain FDAARGOS_748	CP054883.1
	73,858 - 74,556	699	Hypothetical protein	98.71	100	*Staphylococcus hominis* subsp. *hominis* strain K1	CP020618.1
pgcA	74,983 - 76,629	1,647	Phosphoglucomutase	97.63	100	*Staphylococcus equorum* strain KS1039	CP013114.1
gtaB	76,647 - 77,510	864	UTP-glucose-1-phosphate uridylyltransferase	98.15	100	*Staphylococcus xylosus* strain TMW 2.1602	CP066719.1
tagE	77,544 - 79,037	1,494	Poly(glycerol-phosphate) alpha-glucosyltransferase	97.66	100	*Staphylococcus equorum* strain KS1040	CP013114.1
	79,340 - 79,831	492	Hypothetical protein	98.17	100	*Staphylococcus hominis* strain FDAARGOS_762	CP046306.1
cspC	79,973 - 80,173	201	Cold shock protein CspC	99.51	100	*Staphylococcus hominis* strain FDAARGOS_762	CP046306.1
	80,586 - 81,947	1,362	Hypothetical protein	99.12	100	*Staphylococcus hominis* strain FDAARGOS_747	CP046301.1

Table S4 (Continued)

Gene	Position	Size (bp)	Function/ sequence	% similarity	% coverage	Reference strains	Accession no.
yciC	82,241 - 83,440	1,200	Putative metal chaperone YciC	97.6	100	*Staphylococcus hominis* strain FDAARGOS_747	CP046301.1
	83,658 - 83,990	333	Hypothetical protein	97.6	100	*Staphylococcus hominis* strain FDAARGOS_747	CP046301.1
	84,182 - 84,478	297	Hypothetical protein	98.97	99	*Staphylococcus hominis* strain FDAARGOS_136	CP014107.1
	84,709 - 85,221	513	Putative universal stress protein	100	100	*Staphylococcus hominis* strain FDAARGOS_136	CP014107.1
mscS	85,409 - 86,287	879	Small-conductance mechanosensitive channel	100	100	*Staphylococcus hominis* strain FDAARGOS_136	CP014107.1
	86,482 - 86,682	201	Hypothetical protein	100	100	*Staphylococcus hominis* strain FDAARGOS_136	CP014107.1
ISSep*1*	86,976 - 88,799	1,824	IS1182 family transposase ISSep1	99.94	100	*Staphylococcus hominis* strain 19A	CP031277.1
arsC	88,649 - 89,047	399	Arsenate reductase	98.75	100	*Staphylococcus hominis* strain FDAARGOS_136	CP014107.1
	89,087 - 90,634	1,548	Hypothetical protein	99.94	100	*Staphylococcus hominis* strain FDAARGOS_136	CP014107.1
znuA	91,188 - 92,180	993	High-affinity zinc uptake system binding-protein ZnuA	99.9	100	*Staphylococcus hominis* strain FDAARGOS_136	CP014107.1

Table S4 (Continued)

Gene	Position	Size (bp)	Function/ sequence	% similarity	% coverage	Reference strains	Accession no.
	92,590 - 92,366	225	Hypothetical protein	100	100	*Staphylococcus hominis* strain 19A	CP031277.1
brnQ	92,805 - 94,163	1,359	Branched-chain amino acid transport system 2 carrier protein	99.41	100	*Staphylococcus hominis* strain 19A	CP031277.1
	94,300 - 96,420	2,121	Hypothetical protein	99.97	100	*Staphylococcus hominis* strain C34847	CP014567.1
mcrC	96,417 - 97,451	1,035	Protein McrC	98.36	100	*Staphylococcus hominis* strain C34847	CP014567.1
ntaA	97,541 - 98,848	1,308	Nitrilotriacetate monooxygenase component A	99.31	100	*Staphylococcus hominis* strain FDAARGOS_746	CP046306.1
cycA	98,927 - 100,321	1,395	D-serine/D-alanine/glycine transporter	99.64	100	*Staphylococcus hominis* subsp. *hominis* strain K1	CP020618.1
yodC	100,610 - 101,230	621	Putative NAD(P)H nitroreductase YodC	99.68	100	*Staphylococcus hominis* subsp. *hominis* strain K1	CP020618.1
katE	101,440 - 103,449	2,010	Catalase HPII	98.76	100	*Staphylococcus hominis* strain FDAARGOS_748	CP054883.1
cetB	103,697 - 104,227	531	2-epi-5-epi-valiolone epimerase	98.12	100	*Staphylococcus hominis* strain FDAARGOS_746	CP046306.1

Table S4 (Continued)

Gene	Position	Size (bp)	Function/ sequence	% similarity	% coverage	Reference strains	Accession no.
	104,354 - 104,647	294	Hypothetical protein	98.3	100	*Staphylococcus hominis* strain FDAARGOS_745	CP050982.1
lip	104,864 - 107,131	2,268	Lipase precursor/ YSIRK-type signal peptide-containing protein	99.82	100	*Staphylococcus hominis* subsp. *hominis* strain K1	CP020618.1
